# Targeting the Gut: A Systematic Review of Specific Drug Nanocarriers

**DOI:** 10.3390/pharmaceutics16030431

**Published:** 2024-03-21

**Authors:** Patrizia Garbati, Cristiana Picco, Raffaella Magrassi, Paolo Signorello, Ludovica Cacopardo, Mauro Dalla Serra, Maria Grazia Faticato, Maria De Luca, Francesco Balestra, Maria Principia Scavo, Federica Viti

**Affiliations:** 1 Institute of Biophysics, National Research Council, Via De Marini 16, 16149 Genova, Italy; patrizia.garbati@ibf.cnr.it (P.G.); cristiana.picco@ibf.cnr.it (C.P.); raffaella.magrassi@ibf.cnr.it (R.M.); mauro.dallaserra@ibf.cnr.it (M.D.S.); 2 Department of Information Engineering, University of Pisa, Via Girolamo Caruso 16, 56122 Pisa, Italy; paolo.signorello@phd.unipi.it (P.S.); ludovica.cacopardo@unipi.it (L.C.); 3 Research Center ‘E. Piaggio’, University of Pisa, Largo Lucio Lazzarino 1, 56122 Pisa, Italy; 4 Centro 3R: Interuniversity Center for the Promotion of the 3Rs Principles in Teaching and Research, 56122 Pisa, Italy; 5 Pediatric Surgery, IRCCS Istituto Giannina Gaslini, Via Gerolamo Gaslini 5, 16147 Genova, Italy; mariagraziafaticato@gaslini.org; 6 National Institute of Gastroenterology, IRCCS de Bellis, Via Turi 27, 70013 Castellana Grotte, Bari, Italy; maria.deluca@irccsdebellis.it (M.D.L.); francesco.balestra@irccsdebellis.it (F.B.); maria.scavo@irccsdebellis.it (M.P.S.)

**Keywords:** nanocarriers, targeted drug delivery, gut, IBD, IBS, colon cancer, celiac disease

## Abstract

The intestine is essential for the modulation of nutrient absorption and the removal of waste. Gut pathologies, such as cancer, inflammatory bowel diseases (IBD), irritable bowel syndrome (IBS), and celiac disease, which extensively impact gut functions, are thus critical for human health. Targeted drug delivery is essential to tackle these diseases, improve therapy efficacy, and minimize side effects. Recent strategies have taken advantage of both active and passive nanocarriers, which are designed to protect the drug until it reaches the correct delivery site and to modulate drug release via the use of different physical–chemical strategies. In this systematic review, we present a literature overview of the different nanocarriers used for drug delivery in a set of chronic intestinal pathologies, highlighting the rationale behind the controlled release of intestinal therapies. The overall aim is to provide the reader with useful information on the current approaches for gut targeting in novel therapeutic strategies.

## 1. Introduction

The gut is a crucial organ and is in charge of nutrition and evacuation functions. As it represents one of the main barriers separating the human body and the outer environment, it is frequently affected by several pathological states, including cancer, inflammatory bowel diseases (IBD), irritable bowel syndrome (IBS), and celiac disease. Targeted drug delivery can be essential for tackling these diseases [1]. Since its origin in the nineties, the concept of targeted drug delivery has made enormous progress [2]. Nowadays, the aim of targeted therapy is the design of delivery systems that transport drugs to the right site with the right (and hopefully minimal) dose. The most commonly exploited mechanisms to achieve this goal encompass enzyme mediation, pH-dependent release, the use of special vehicles, receptor targeting [3], and the recent microbiome-targeting approach [4], among others. Conceptually, targeted delivery strategies include ‘active targeting’ and ‘passive targeting’. The first is based on the principle of ligand–receptor binding, which relies on the specificity of the ligand for the receptors expressed on the surface of the target cell. This mechanism often depends on the blood circulation and extravasations, and it can take place only if the carriers and the target site are adjacent. Passive targeting does not rely on specific ligand binding to the target site; instead, it exploits, for example, enzyme activity and pH variations [3]. Over the last decade, functional biomaterials research has fostered the development of new advanced drug delivery systems. In particular, nanotechnologies offer innovative approaches to improve the effectiveness of drug treatments. Natural or synthetic nanocarriers were proven to be able to deliver drugs directly to the desired sites, improving the precision of the release and overcoming the off-target activities of the drugs. Moreover, carriers should protect the incorporated drugs from undesired enzymatic attack. Finally, nano-encapsulated systems should allow the controlled release of drugs over time, ensuring a longer duration of action and reducing the need for frequent dosing [5]. Nanocarriers are widely used to target the intestinal tract, effectively supporting drug delivery in these tissues and improving the efficiency of the released treatment.

**Figure 1:**
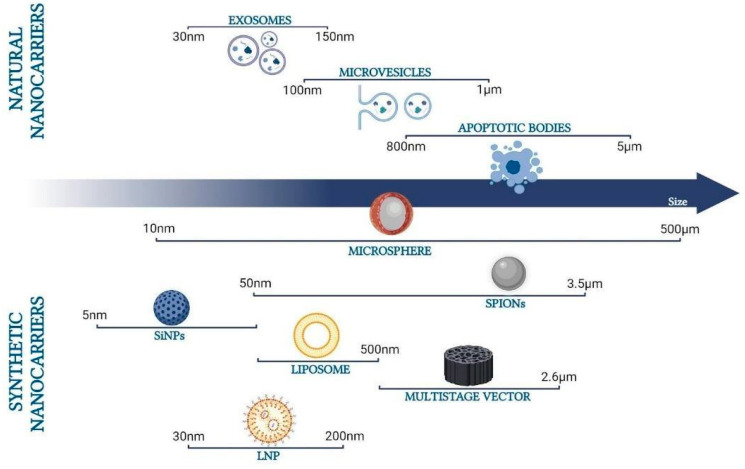
Schematic overview of nanocarrier size.

**Figure 2:**
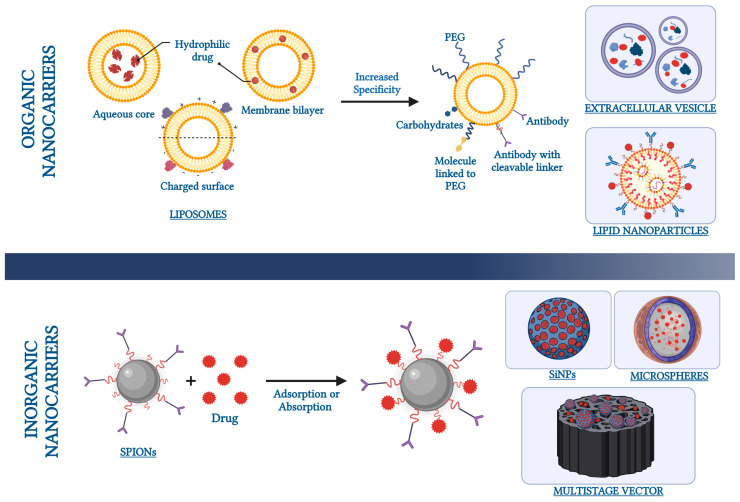
Simplified schema of composition of organic and inorganic drug nanocarriers. In the upper panel (organic nanocarrier), liposomes appear modified with molecules, such as PEG + functionalizing layer, antibodies with cleavable sites, carbohydrates, and hydrophobic/hydrophilic drugs. Other organic carriers are reported (EVs and lipid nanoparticles). Inorganic nanocarriers (lower panel) can be modified with antibodies and/or drugs. In particular, drugs can be contained inside (such as in microspheres) or linked outside (such as in SiNPs) the carrier. Moreover, modifications can adhere to the surface of the nanoparticles by means of surfactants or they could be packaged in the pores (such as in the case of multistage vectors).

In the following, a systematic review of the literature is carried out. The aim is to provide readers who are approaching the field of drug delivery systems targeting the intestine with a comprehensive overview of the current strategies that are used to specifically release pharmaceuticals at this site. To this end, the most recent (in the last 5 years) publications concerning the nanocarriers that are able to selectively release formulations to the gut were selected and analyzed. Different types of carriers were identified (Figure 1), including natural and synthetic carriers that are able to reach the intestinal tract affected by highly impactful diseases: inflammatory bowel diseases, colon cancer, celiac disease, and irritable bowel syndrome. The considered nanocarriers encompass structures of different sizes and compositions (Figure 1 and Figure 2) and are typically exploited in the context of gut targeting. These include lipid-based nanoparticles (such as in the case of liposomes or nanostructured lipid carriers), inorganic nanoparticles (such as in the case of SPIONs), and polymeric nanoparticles (such as in the case of natural alginate and chitosan, but also PLGA). Some of the materials present features which directly enable gut targeting, others represent ideal carriers in terms of biocompatibility, robustness, and capacity but need to be functionalized to specifically release content to the gut. These aspects are treated in detail in the text. In order to be able to include most of the literature available in this context, specific nanocarrier-related keywords were used to enable the online search, including SPIONs, liposomes, silica, extracellular vesicles, NLCs, SEDDS/SMEDDS/SNEDDS, and microspheres. At the end of the review, critical considerations regarding the approaches enabling gut targeting are reported.

### 1.1. Gut Structure

**Figure 3:**
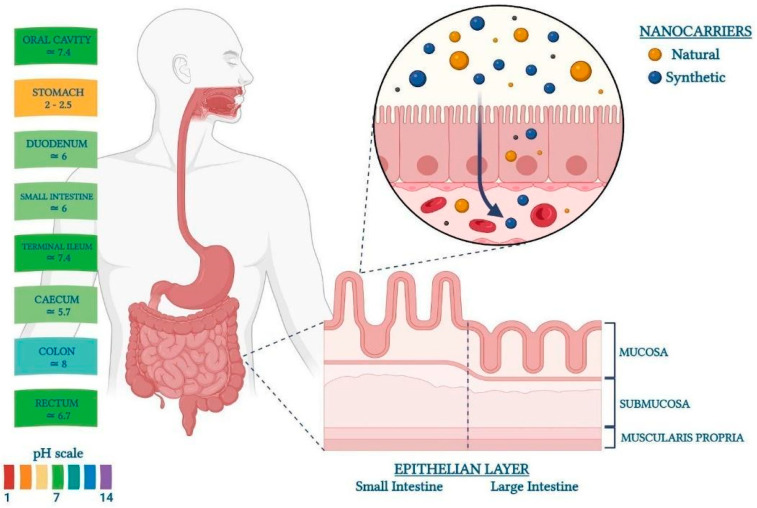
Illustration of pH variants in the intestinal tract, with specific focus on the structure of the large and small intestines.

Digestion and absorption of nutrients, secretions, and immune response are the main functions of the gut, together with the possibility of freeing the organism from the waste material. To accomplish this set of crucial tasks, the intestine relies on a complex structure. The human adult intestinal system is over 9 m long and consists of the small intestine (approximately 7 m) and large intestine (around 2 m) [6]. The overall length of the gut is adequate to guarantee the correct nutritional function of the individual. In particular, the small intestine (composed of the duodenum, jejunum, and ileum) is assigned the task of breaking down food, mixing it, and absorbing the nutrients needed for the body [7]. The large intestine, formed by the cecum, colon, rectus, and anus, has the main function of absorbing water and electrolytes and eliminating feces [8]. The process of propagating food from the proximal to the distal end of the gut, allowing nutrient absorption and waste propulsion, consists of two harmonized movements: peristalsis (or mass movement), which consists of strong contractions that serve to move the chyme to the rectum quickly, and segmentation (or haustral contraction), which allows the slow movement of food while mixing the chyme to promote absorption. Gut movements are ascribable to the intestinal smooth muscle, which is organized into an inner circular and an outer longitudinal layer. Between these two muscle layers lies the myenteric plexus, a network of nerves containing the interstitial cells of Cajal (ICC). Other crucial functions are carried out by the gut, which is responsible for absorbing food components (absorptive function) and also represents the border that separates the external environment (materials contained within the gut lumen) from the body, thereby acting as a barrier that protects the organism from the invasion of foreign contaminants (barrier function) [9]. To carry out the whole set of functions, the wall of the intestinal tract is equipped with four layers: the mucosa (epithelium, lamina propria, and muscular mucosae), which is mainly responsible for the absorptive and barrier functions; the submucosa; the muscularis propria (inner circular muscle layer, intermuscular space, and outer longitudinal muscle layer), which is in charge of gut motility; and the serosa. The intrinsic differences between the tissues and functions in the gut are guaranteed by the localization of distinct cell types, the intricate transcriptional programs governing individual cells, and the interaction with other systems (such as the enteric nervous system, which orchestrates muscle contraction). Another interesting aspect of the gastrointestinal tract is the intrinsic pH variation (Figure 3). Along the gastroenterological tract, the pH fluctuates between 7.0 (in the oral cavity) and 8.0 (in the colon). In detail, the intraluminal pH is highly acidic in the stomach, reaching pH 6 in the duodenum. Then, it gradually increases through the small intestine, measuring pH 7.4 in the terminal ileum. It drops to 5.7 in the caecum and then gradually increases again to pH 6.7 in the rectum [10].

### 1.2. Highly Impactful Gut Pathologies

In this complex anatomical and functional scenario, several pathologies can affect the organ and its functions. Basically, diseases of the gastrointestinal tract can be classified into two categories: functional and structural. Functional gastrointestinal diseases are a group of disorders described by chronic gastrointestinal symptoms (e.g., abdominal pain, dysphagia, dyspepsia, diarrhea, constipation, and bloating) without evident pathology [11]. In contrast, the structural diseases of the gut include diverticular disease, stenosis, hemorrhoids, strictures, inflammatory bowel disease, colon polyps, and colon cancer [12]. The origin of gut diseases could be genetic (somatic or germline); autoimmune (celiac disease, Crohn’s disease, or ulcerative colitis); infective (gastritis or gastroenteritis); morphological (malformations); or environmental (i.e., related to lifestyle, feeding habits and stress). Among the gut pathologies which have the greatest impact on patients’ lives, a subset was chosen for purpose of this review; this subset represents a major burden for patients and society, and the scientific community is insistently looking for therapeutic strategies to threat these pathologies: inflammatory bowel diseases (IBD), colon cancer, celiac disease, and irritable bowel syndrome (IBS).

IBD is a group of chronic inflammatory conditions that affect the gastrointestinal tract. IBD is believed to result from a complex interplay between genetic and environmental factors, leading to dysregulated immune responses. The two primary types of IBD are ulcerative colitis (UC) and Crohn’s disease (CD), each of which is characterized by distinct features [13]. It has been estimated that IBD impacts up to 7 million people worldwide [14]. The main symptom of these chronic inflammatory conditions is pain, a manifestation which is present in about 50–70% of the affected patients [15]. Other possible symptoms are dysphagia, abdominal bloating/distension, change in bowel habits, rectal bleeding, and dyspepsia [16]. This condition significantly diminishes patients’ quality of life, primarily because of the elevated recurrence rate of symptoms and the necessity for lifelong care [17]. Different types of synthetic and natural nanocarriers have been developed to target IBD conditions.

Another common chronic disorder affecting the intestinal tract is IBS. It is characterized by abdominal pain associated with altered bowel movement and often bloating in the absence of morphological changes [18]. The cause has not yet been elucidated, but multiple factors contributing to its onset are known (i.e., altered gastrointestinal motility, visceral hypersensitivity, post-infectious reactivity, brain–gut interactions, alteration in fecal microflora, bacterial overgrowth, food sensitivity, carbohydrate malabsorption, and intestinal inflammation) [18].

A recurrent pathology of the gut is cancer, which rarely affects the small bowel but has high incidence in the colon. Colorectal cancer (CRC) is the third most common diagnosis and the second deadliest malignancy in the United States. It could present as sporadic, familial clustering and, in a few cases, inherited syndromes. The principal prognostic colon cancer indicator consists of the pathological stage at diagnosis. Resection, when feasible, and adjuvant therapy represent the most recurrent treatments [19]. However, most chemotherapeutics are non-specific for tumors and induce severe adverse effects. Therefore, the use of targeted drug delivery systems represents a promising strategy with which to discriminate between tumor and healthy tissue.

Celiac disease, a condition caused by an interplay of environmental, genetic, and immunologic factors, is characterized by small intestinal mucosal injury in genetically susceptible individuals when ingesting gluten. The spectrum of disease can vary markedly among affected subjects and can result in total villous atrophy, which is responsible for severe diarrhea and malabsorption [20]. Celiac disease prevalence is set at between 1.8% (a probably overestimated value retrieved from serological studies) and 0.7% (a probably underestimated value obtained from biopsy-diagnosed studies), and it seems to consistently increase over time across different geographical regions [21].

It is worth noting that some gut pathologies, such as IBD and cancer, appear to be characterized by the so-called “epithelial enhanced permeability and retention” (eEPR) effect, an enhanced accumulation of compounds in the affected tissues, which might be ascribed to the disordered nature of the mucosal barrier (impairment of epithelial tight junctions, and/or the immigration of immune cells) in the presence of this pathological condition. This scenario seems to intrinsically promote nanocarrier penetration in intestinal tissues [22,23]: nanocarrier local uptake and selective binding can take advantage of the heightened intestinal permeability (100 nm–2 µm junctions compared to normal 2–6 nm tight junctions) and the alterations in electrostatic interactions at the site of intestinal inflammation [24], promoting particle passive accumulation in the gaps between the cells [25] and avoiding their rapid elimination by diarrhea.

The accelerated transit (as a consequence of diarrhea) is another feature typically associated with gut pathologies. In this case, formulations that increase the local drug concentration and residence time in the tissue can represent an effective strategy for therapy delivery.

Finally, a condition often coupled with gut diseases is related to intestinal mucus layer thickness, which tends to be increased in inflamed/ulcerated regions, possibly allowing mucus-adherent nanoparticles to localize in these regions.

## 2. Materials and Methods

### 2.1. Nano- and Micro-Carriers

Drug delivery commonly occurs through canonical routes, which encompass oral/buccal, rectal, subcutaneous, intranasal, intramuscular, intravenous, pulmonary, and transdermal administration. These traditional methods present ascertained limitations, such as the risk of delivery displacement accompanied by potentially severe side effects, uncontrolled release, and enzymatic deterioration of the formulation. The incorporation of drugs into nanocarriers is an efficient strategy for the targeted and sustained delivery of drugs [1]. Nanocarriers are defined as systems, with at least 1 dimension below 100 nm, that are able to incorporate drugs into organic or inorganic matrices [26]. According to the European Union Observatory for Nanomaterials (EUON), a nanomaterial can be any “natural, incidental, or manufactured material containing particles, in an unbound state or as an aggregate or as an agglomerate and where, for 50% or more of the particles in the number size distribution, one or more external dimensions is in the size range 1–100 nm” (https://euon.echa.europa.eu/definition-of-nanomaterial (accessed on 20 December 2023)). More specifically, the ISO guidelines define a nanoparticle (NP) as an object with 3 dimensions below 100 nm (https://www.iso.org/obp/ui/en/#iso:std:iso:80004:-1:ed-1:v1:en (accessed on 20 December 2023)). Nanotechnology has emerged as an expansive domain in biomedical applications, showcasing diverse nanocarriers like polymeric nanoparticles, superparamagnetic iron oxide nanoparticles (SPIONs), silica particles, and others. These carriers exhibit significant physical and chemical properties, leveraging the advantages of their nanosized effects to guarantee, in drug delivery systems (DDS), bioavailability and minimized side effects [27,28]. Moreover, nanocarriers can be formulated to have specific surface properties targeting the desired cells, further enhancing drug efficacy and reducing side effects [29]. SPIONs are classified as inorganic carriers [30] and are used most extensively as antibacterial agents due to their unique physical, chemical, magnetic, and biocompatibility properties. Despite their ferromagnetic nature, the nanoscale dimension provides the particle with superparamagnetic features: they magnetize only in response to an external magnetic field because of the combination of Brownian and Neel effects [31]. In addition, the small size of these particles makes it possible to form aggregates by dipole–dipole interactions and to move according to magnetic field lines [32]. Another type of nanocarrier commonly used in biomedical applications is based on silica nanoparticles (SiNPs), which are composed of silicon dioxide and are characterized by controllable particle size, large surface area, and great biocompatibility. Silica is a material that has been approved as safe by the Food and Drug Administration (FDA) (https://www.cfsanappsexternal.fda.gov/scripts/fdcc/index.cfm?set=FoodSubstances&id=SILICONDIOXIDE (accessed on 20 December 2023)). SiNPs have been widely described as drug carriers and recognized for their biocompatibility characteristics [33], although the toxicity mechanisms of these materials are poorly understood [34]. The possible toxicity is particularly highlighted, in a dose-dependent manner, when SiNPs are administered in animal models, where exposure to SiNPs results in inactivity, nervousness, and ultimately mortality [35], along with intestinal inflammation [34]. The drugs are typically loaded either by incorporating them during nanoparticle production or by adsorption/absorption after nanoparticle formation [36]. A type of nanoparticle widely studied for drug delivery in the gut is represented by the multistage silicon-based nanoparticles, a drug delivery platform for several drugs, such as sulindac and silymarin, that preferentially interacts with colon cancer cells as opposed to normal intestinal mucosa [37]. Among the typical nanocarriers, there are also lipid-based nanoscale structures [30]. The interest in lipid-based nanocarriers for drug delivery in the gut is due to their ability to improve the stability, solubility, and permeability of their cargo drugs [38]. Liposomes and lipid nanoparticles (LNPs) belong to this category of compounds. Both are excellent drug delivery vehicles that transport the cargo of interest within a protective outer layer of lipids. They are similar in appearance but different in terms of inner structure, composition, and function. LNPs are specifically designed to encapsulate a wide range of nucleic acids (RNA and DNA), making them the most favored non-viral system for gene delivery. LNPs stand out as highly successful nano-delivery vehicles that facilitate the efficient delivery of cytotoxic chemotherapy agents, antibiotics, and nucleic acid therapeutics [39]. Liposomes are spherical lipid vesicles (with particles sizes that are usually 50–500 nm in diameter) composed of one or more lipid bilayers with an aqueous core, which is a result of the emulsification of natural or synthetic lipids in an aqueous medium. Liposomes are considered to be promising drug nanocarriers for various hydrophobic and hydrophilic molecules due to their high biocompatibility, biodegradability, nanosized-specific properties, and low immunogenicity [40]. They can encapsulate drugs either in the aqueous interior of the vesicles or in the lipophilic membrane, thereby protecting therapeutic molecules from degradation and clearance and sustaining their release for longer times [41]. Liposomes act as stabilizing compounds, overcoming barriers to cellular and tissue uptake, improving drug distribution to specific target sites, and minimizing systemic toxicity [42]. The conventional liposomes lack stability during storage and are weak in active targeted absorption in the gastrointestinal tract (although they can accumulate at the pathological site because of the eEPR effect). At present, surface modification has been approved as an effective platform to shield barricades and help liposomes deliver the agents safely and effectively to the ideal site [43]. Examples of surface modifications of liposomes can include the use of polymers+functionalizing layers, antibodies with cleavable sites, carbo-hydrates, hydrophobic/hydrophilic drugs (Figure 2), peptides, aptamers, or other small molecules, all of them facilitating the targeting of specific body regions [44]. Moreover, liposomes, such as other nanocarriers, can contain elements that, when coupled with physical stimuli, enable the drug release at the desired time and/or site. This can be the case with temperature, magnetic field, or laser irradiation; taken alone, or sometimes joined together, these physical–chemical approaches allow further control in therapy release [44]. Although liposomes can be administered either intravenously or intramuscularly, novel methods are emerging to enable oral administration, to protect carriers and drugs in the harsh gastrointestinal environment. Thus, the phospholipid bilayer can be ‘PEGylated’ (i.e., coated with polyethylene glycol polymers) [45]. Such liposomes can be easily absorbed by the enterocytes, allowing them to reach the circulatory system. PEG also increases the stability of liposomes in the blood, improving both their circulation and their drug release time [46,47,48]. A novelty in the field is represented by nanostructured lipid carriers (NLCs), a second generation of lipid carrier developed as an alternative to polymeric nanoparticles, liposomes, solid lipid nanoparticles, microparticles, and emulsions [49]. Usually, the NLCs are composed of lipids (solid or liquid), surfactants, counter-ions, and emulsifying agents and are chemically and physically stable systems with improved drug incorporation and increased bioavailability [50]. The NLCs are used especially to improve the bioavailability, cytotoxicity, and uptake at a specific site.

An option for hydrophobic/lipophilic drug delivery through oral administration is represented by self-emulsifying drug delivery systems (SEDDS), also known as SNEDDS or SMEDDS to indicate their nano (N) or micro (M) size. They consist of mixtures of oils, co/surfactants, and co/solvents that form isotropic blends and are often administered through gelatin capsules. SEDDS/SNEDDS/SMEDDS systems can improve the bioavailability of drugs [51] and protect them from precipitation and premature degradation [52]. Upon contact with gastrointestinal fluids and gentle agitation, these systems undergo aqueous dilution, resulting in the formation of fine and relatively stable oil-in-water emulsions, which enhance the overall hydrophobic drug absorption in the gut [53], especially when they are coupled with gut-targeting compounds.

In the last decade, numerous studies have shown the possibility of using modified extracellular vesicles (EVs) as natural nano-vectors containing drugs directed to a specific site. EVs are membranous vesicles derived from cells; they encapsulate diverse biological molecules, such as proteins, lipids, and genetic material [54]. The use of modified EVs allows an increase in therapeutic efficacy due to a greater specificity linked to cell-specific targeting [55,56,57]. EVs are vesicles derived from most cell types and are classified on the basis of their size: exosomes (30–150 nm) (EXO); microvesicles (100–1000 nm) (MVs); and apoptotic bodies (800–5000 nm) [58] (Figure 1). They are distributed throughout biological fluids [59] and are involved in several patho-/physiological processes, which are specifically exploited during cellular crosstalk [60]. Many studies have highlighted the differences between EVs secreted by normal and cancer cells in terms of size and composition. Such variations are principally due to the characteristics of the microenvironment. In particular, cancer cells produce a higher amount of EVs, and their secretion is related to the peculiar features of the tumor milieus, such as oxidative stress, pH gradients, nutrient deprivation and competition, hypoxia, increased interstitial pressure, and growth factor release [61] (Figure 2).

Finally, an interesting approach to controlled drug release is represented by microspheres. Microspheres refer to a broad class of particles with diameters ranging from the high nanometer scale to the micron scale; they exhibit a spherical shape, typically between 1um and 100 um in diameter. Microspheres find applications in various biomedical activities, particularly in controlled drug release applications. In contrast to nanoparticles, for which small dimensions offer an additional factor that promotes drug uptake, microspheres have been used as drug delivery systems mainly to protect the drug and to improve its local delivery [62]. The most prevalent types include solid and hollow glass microspheres, solid and hollow polymer microspheres, and ceramic microspheres. These microspheres can be crafted from a variety of raw materials, including polymers, glass, cellulose, silica, and metal.

The adequateness of these carriers in delivering drugs specifically to the gut is discussed in the present review.

### 2.2. Literature Search Strategy and Study Selection Process

**Figure 4:**
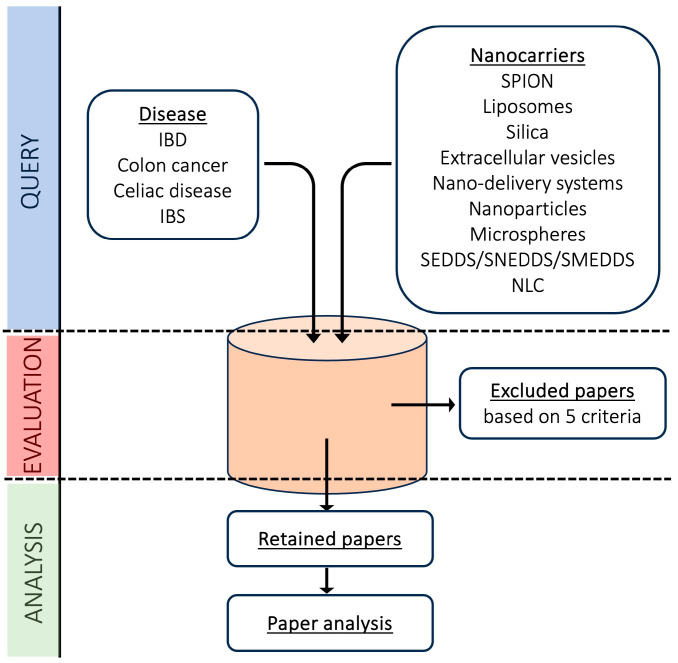
Overall strategy implemented in this review.

The systematic review was carried out according to PRISMA guidelines, and the overall strategy is described in Figure 4. The searches were conducted in November 2023 on the Medline PubMed online database and referred to papers published in the last 5 years (2018–2023) in order to provide an up-to-date overview. The literature search was carried out by applying a set of keywords which represented the inclusion criteria of this study selection process. The exploited queries derived from the combination of several pathological states of the gut (‘Inflammatory bowel diseases’, ‘Colon cancer’, ‘Celiac disease’, and ‘irritable bowel syndrome’) and the most consolidated nanocarriers (‘Nanoparticles & Drug delivery’, ‘Liposomes’, ‘Vesicles’, ‘Microspheres’, ‘NLC’, and ‘SEDDS/SNEDDS/SMEDDS’).

In order to include in the selection the specific nanoparticle-based approaches which are neglected by the ‘Nanoparticles & Drug delivery’ generic search, queries were also carried out using specific nanoparticle-related terms: ‘SPION’, ‘Silica’, and ‘Nano-delivery systems’. Abstract analysis was performed on the queried results, applying the exclusion criteria, which allowed the following to be left out: reviews and books (criterion 1); papers where the full text was not freely available (criterion 2); duplicated results (criterion 3); and non-English papers (criterion 4). A further coarse-grained text overview was performed, with 2 independent reviewers for each query, to exclude articles not relevant to the topic of the review (criterion 5), while keeping in mind that the manuscript focuses on nanocarrier approaches to specifically target the gut in drug delivery. The final set of papers underwent a fine-grained manual data extraction process to highlight, for each publication, the exploited/developed nanocarrier type, the model where it was tested, and, most importantly, the rationale underlying its ability to release the transported drug selectively into the gut. The present systematic review was not registered on the PRISMA-compliant databases.

## 3. Results

### 3.1. General Overview of the Selected Papers

**Figure 5:**
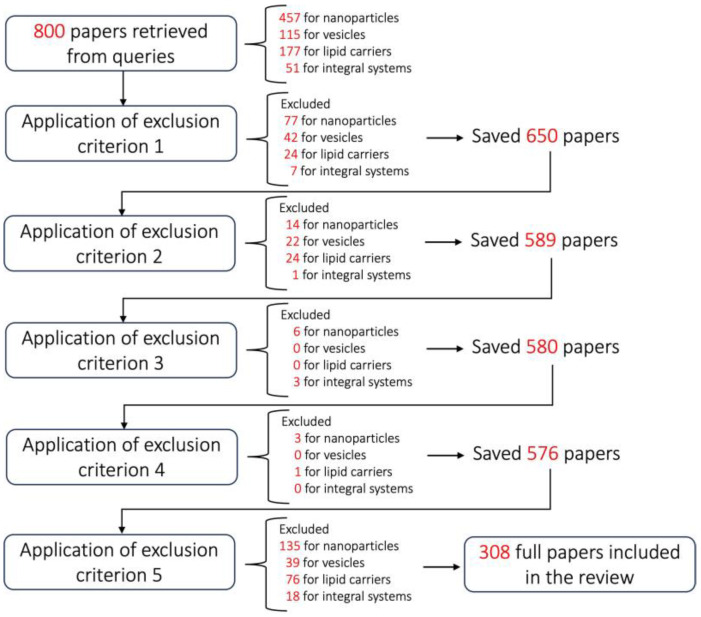
Flowchart of the inclusion/exclusion process leading to the final number of analyzed papers. Integral systems include microspheres and SEDDS/SNEDDS/SMEDDS.

The initial paper search yielded 800 results. After applying the exclusion criteria, 308 articles were confirmed to contribute to the present review. The flowchart in Figure 5 provides the details of the article exclusion process.

The initial and final amounts of papers for each literature search are reported in Table 1; they are classified on the basis of the queried nanocarrier and disease.

**Figure 6:**
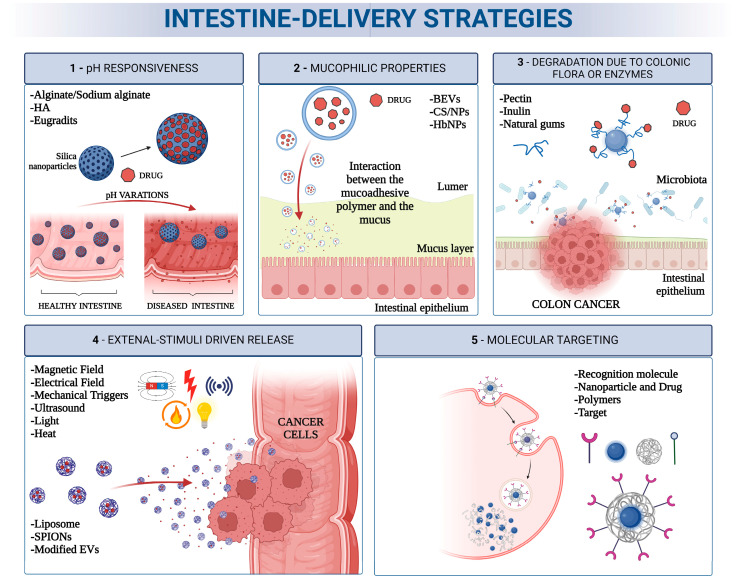
Summary of intestine delivery strategies.

At the end of the process, the highest number of results was retrieved for the IBD and colon cancer pathologies, which are in fact among the most attractive intestinal pathologies for the scientific community studying novel therapeutic solutions, which are also mediated by nanocarriers. The oral delivery mode appeared to be the most common administration route for the gut due to the natural passage into the intestine which is intrinsic to this approach. In order to provide a critical and usable overview of the results, the gut-targeting strategies were classified on the basis of the mechanism they activated or exploited to release the compound in the desired region (Figure 6) or to enhance its uptake in tissues and cells; thus, this classification resembles and extends the classification proposed by Ibrahim and colleagues [63].

### 3.2. pH Responsiveness Properties

The most exploited strategy to deliver drugs to the colon is probably that which uses pH-sensitive nanocarriers, which are characterized by their ability to alter their physical properties in response to the specific pH levels of the external environment. This approach relies on the peculiar feature of the gut related to acidity, which ranges from approximately 2–2.5 in the stomach to 8 in the colon [64]. The extremely acidic pH of the gastric tract, together with other physiological factors, such as a high intestinal enzyme activity, may be responsible for the drug degradation before they reach the site of interest in oral administrated therapies. pH-responsive materials allow the preservation of the drug carried by the nano-cargo, exploiting this environmental responsivity as a ‘driving force’ for controlled drug release to the gut. pH-triggered release is also considered to be a selective indicator for precise drug release in the case of tumors. In fact, in such conditions the slightly acidic tumor microenvironment generated by the Warburg effect could lead to a drop in the pH to around 5.5, with cancerous cells and tissues being more acidic than the healthy microenvironments. Thus, smart pH-sensitive nanocarriers have been engineered to target colon pathologies, especially through oral drug administration, in the context of IBD and colon cancer, enhancing the specificity and effectiveness of treatments [65,66,67,68,69,70,71,72,73]. In particular, polyacid polymers, such as alginate and hyaluronic acid, are intrinsically suited for delivery in the gut; at an acidic pH, they can be found in their neutral shrunken form, protecting the drug during the passage through the stomach. In contrast, when the pH is higher than the acid dissociation constant (pKa), they assume an ionized form, which promotes electrostatic repulsion among the polymer chains and gel swelling. Alginate, a polysaccharide from seaweed, is used in drug delivery systems, either alone or in combination with other molecules, due to its characteristics of hydrophilicity, biocompatibility, biodegradability, safety, ease of use, and ionic crosslinking [74]. Alginate possesses the ability to swell, which facilitates time-dependent drug release [75]. Nanocarriers based on disulfide bond-containing polymers (such as sodium alginate) are stable in normal cells. On the other hand, they degrade in cancer cells due to their reductive environment (low redox potential due to the higher concentration of glutathione in cancerous cells than in normal ones), thus allowing drug release only at the desired site and reducing toxicity and side effects. As a polyacid, alginate is particularly suited to the protection of therapeutic compounds from the acidic gastric environment, while ensuring their release at higher pH levels in the colon [76,77], given its pKa of around 3.5–4.6 [78]. Moreover, alginate presents a mucophilic property that improves intestinal adhesion, thus promoting contact between the released drug and cells and the related absorption. In fact, mucins contain positively charged regions (Ca^2+^), which might foster the binding of alginate (or its compounds) through electrostatic and hydrogen bonding [79]. Arif et al. [80] used thiolated alginate NPs for colon-specific drug delivery in IBD, showing an increase in drug release at pH 7.4, thus indicating their adequacy for colon-specific drug delivery [81]. Alginate microspheres were exploited for drug delivery in an IBD [82] and IBS context; for example, Zhang H.Y. et al. [83] proposed the use of an alginate shell to coat a chitosan and thioketal core in order to obtain microspheres able to protect puerarin against destruction in the gastrointestinal tract, while allowing its release at the lesion site. In the same IBS context, alginate microbeads were proposed to microencapsulate the therapy; they consisted of peppermint oil, which is characterized by an instant and short duration effect and by instability in unfavorable environmental conditions, such as in the gastric tract [84]. In in vitro conditions, when immersed in a pH 6.8 solution, the alginate microbeads began to swell and erode, thus releasing the drug. Alginate-based NPs were also used in the context of colon cancer therapy [85,86,87,88,89,90,91]. Even in the field of EV-based therapy, stomach acidity represents a limiting factor in oral therapy. For this reason, EV modifications have been exploited. In Liu et al.’s work [92], EVs were first conjugated to galactose to protect the drug from degradation in the stomach; then, a chitosan/alginate hydrogel was used to coat the EVs to ensure a favorable delivery to the colonic tract. Similarly, hyaluronic acid (HA) and its conjugates have been used to formulate nanocarriers for IBD treatment [93,94,95] and for colon cancer drug delivery [96,97]. HA is an unbranched glycosaminoglycan component of the extracellular matrix. The NPs produced that include HA show several useful features, such as non-immunogenicity, biosafety, and anti-inflammatory activity [98]. Wei et al. [99] described HA-modified L-arginine CO_2_ NPs loaded with Pterostilbene. Under lysosomal pH conditions, HA-PS@NPs liberate CO_2_ and generate a pH-activated nano-bomb effect, which augments the cytosolic delivery of the drug in ulcerative colitis; this has also been confirmed in in vivo tests in mice. In another study, HA-functionalized porous silicon NPs were developed to link an enzyme-responsive hydrogel and a pH-responsive polymer [100]. This nano-system provided a ‘hierarchical structured vehicle’ of different porous NPs and stimuli-responsive materials, enhancing drug delivery to IBD-affected gut cells. Several HA-functionalized nano-delivery systems were also designed for colon cancer treatment, including functionalized liposomes [101], cubosomes [102], zinc oxide [103], and iron oxide nanocomposites [104]. Often, they exploit the HA affinity for CD44, which is highly expressed in tumor cells. Salimifard et al. [105] and Esmaily et al. [106] developed nanoparticles by combining HA with chitosan and lactate to target the colon and impede tumor progression. Encapsulation in NLCs coated with HA and mPEG allowed a reduction in the toxicity of cantharidin [107], an active compound from *Mylabris cichorii*, which was promising for colon cancer therapy, but its toxicity in oral administration limits its clinical application [108].

Another interesting pH-responsive nanocarrier for the therapy of intestinal diseases is Eudragit. Eudragits are non-biodegradable, nonabsorbable, and nontoxic polymethacrylate-based copolymers, which are present on the market in different formulations for the targeting of different sections of the intestinal tract. ES100 is an anionic copolymer of methacrylic acid and methyl methacrylate (1:2 ratio). It is often used as an excipient because it enables a pH-sensitive dissolution at about pH 7.2, allowing a specific release of the drug into the colon [109]. ES100 and ERS100 (a time-dependent polymer) were extensively used in targeted oral drug delivery systems; they were used singularly or coupled together [110,111] and even in the form of microspheres [112] and showed an effective response to pH changes along the gastrointestinal tract. They were exploited for IBD [113], IBS [114,115,116], and colon cancer treatments [117]. Eudragits can be coated with biopolymers such as chitosan to increase the adhesion to intestinal mucosa. This solution has been exploited to deliver drugs in the presence of colon cancer [63,118,119,120] and IBD [121]. As a multi-functionalized oral nano-delivery system based on the combination of HA with a polylactic acid–glycolic acid copolymer (PLGA), Eudragit^®^ S100 (ES100) and chitosan [122] have been developed for IBD treatment. In this case, the administered drug was Methotrexate (MTX), an immunosuppressive agent which caused systemic immunosuppression in its in vivo application [123]. Interestingly, MTX-loaded HA-CS/ES100/PLGA NPs showed colon-specific activity in a mouse model. HA/CS/ES100-based approaches were also used to deliver other drugs [124,125]. Eudragit L100 and S100 were used to encapsulate SPIONs for the oral administration of colon cancer drugs [126]. The compound underwent pH-responsive dissolution in in vitro tests, showing that the formulation could rapidly release the drug (carmofur) at pH 6.5 and 7.4, while protecting it from digestion in acidic environments. Eudragit^®^ FS 30D, a pH-sensitive polymer that dissolves at pH values above 7.0, was assembled in microspheres for IBD targeting, coupled with sodium alginate and inulin [127]. The latter, an oligosaccharide that resists degradation in the stomach and small intestine, can be broken down by enzymes produced by resident colonic bacteria, thus allowing drug release. Eudragit was also used to engineered NLCs for the colon targeting of 5-Fluorouracil; its effectiveness in terms of higher bioavailability and longer release was confirmed in both in vitro and in vivo (in rats) tests [128]. Although less exploited, other molecules exist which can intrinsically enable pH sensitivity in a formulation. Carboxymethyl starch (CM) is a pH-responsive excipient used as a drug gastro-protector due to the presence of carboxyl groups on its starch chains [129]. A combined release system was developed based on the integration of LDHs into CM microspheres [130] in order to improve colon cancer therapy delivery. Succinic acid and the succinyl group are natural compounds exploited for their mucophilic properties [131,132]. Cholesteryl hemisuccinate (CHEMS) is widely used to provide liposomes with pH sensitivity because of its lipophilicity and membrane stabilization activity. At alkaline and neutral pH, it self-assembles into a bimolecular layer and fuses with liposomes, and it dissolves as soon as it comes into contact with the tumor acid environment, facilitating drug release [133]. Layered double hydroxides (LDHs) are cationic layered compounds with exchangeable anions between the layers, which enable the drug interlayer loading and pH-responsive release [134]. To facilitate the programmable release of quercetin, Yilmaz M. et al. (2019) exploited the pH condition which is typical of tumors by coating gold nanoparticles with calixarenes, which present pH-responsive properties [135]. Another pH-dependent release strategy includes functionalization with listeriolysin O (LLO) [136]. LLO is part of the cholesterol-dependent cytolysin family produced by the bacterial pathogen *Listeria inside* during infection of eukaryotic host cells. The protein self-assembles at low pH, promoting the formation of pores in the endosomes, thus enabling endosome escape [38]. A new pH-responsive nano-delivery system is ZIF-8 [137], whose NPs showed high loading capacity and positive charge and demonstrated that they were good drug carriers. Moreover, its pH-sensitive behavior allowed this nano-delivery system to guarantee the release of the loaded antibiotic (imipenem) at the acidic infection site.

pH sensitivity can also be derived from the carriers’ functionalization. This is the case with silica mesoporous microparticles [138], which are carrier structures showing promising results in both ulcerative colitis and colon cancer cells [139,140]. A silica-containing antioxidant nanocarrier (siRNP) was developed to ameliorate the absorption of hydrophobic drug molecules and to enhance the stability of nanoparticles in the gastrointestinal environment [141]. The coating of mesoporous silica nanoparticles with various molecules, such as (3-Glycidyloxypropyl) trimethoxysilane (3GPS), can improve drug delivery and cancer cell targeting, as demonstrated in the work by Asiri et al. [142]. Other solutions foresee the use of alginate-coated mesoporous silica nanoparticles in colon cancer therapy [143,144]. Yang et al. developed mesoporous silica nanoparticles coated with HA to enhance the colon targeting that relies on HA pH responsivity [145,146]. Another interesting approach was reported by Broesder et al., who used SEDDS in colon cancer therapy to administer a lipophilic drug. In order to enhance drug release in the gut, they combined SEDDS with a pH-sensitive coating technology (ColoPulse), which improved drug dispersion in the colon [147].

Electrostatic interactions between nanoparticles were exploited by Qiu et al. [148] to deliver drugs in colorectal cancer tissues. The proposed system was based on PEG, which protects NPs from enzymatic damage, and glutamic acid, the functional group that provides electrostatic interaction. The environmental acidity of cancer has an impact on the surface charge of the formulation and affects the electrostatic interaction between the nanoparticles and the drug, thus resulting in drug release in the targeted environment.

To summarize, gut targeting for drug release driven by pH sensitivity involves a set of carriers that, in oral delivery, can resist the harsh conditions of the stomach (Figure 3), while being dissolvable at the basic pH levels of the intestine (Figure 6(1)). This feature is typical of compounds such as alginate, HA, Eudragits, and calixarenes. In particular, the natural origin of the first two polymers presents advantages in terms of environmental sustainability, wide availability, and low cost. They also have an intrinsic biocompatibility and mucophilicity, which are crucial properties for gut delivery. On the other hand, Eugradits and calixarenes, being of synthetic origin, need to be further functionalized to acquire the ability to interact with intestinal mucosa. However, they may show higher stability in the physiological environment with respect to natural polymers, which could undergo partial degradation by enzymes and intestinal bacteria.

### 3.3. Nanocarrier Mucophilic Properties

Affinity with the intestinal mucosa represents another important feature when designing strategies for targeted delivery to the gut. Mucoadhesion is one of the most relevant interactions between mucin glycoproteins and biopolymers. It can reduce the diffusion pathway of drugs, protecting them from enzymatic activity and luminal degradation and ultimately improving drug efficacy by maximizing its absorption [149]. Also, mucophilic properties are essential to increase the retention time of the carrier in the intestine and to improve the availability of drug following diffusion from the swelled carrier at high pH levels. These processes mainly rely on polymer diffusion inside the mucus layer and on the entanglements and electrostatic interaction with mucins [150]. In detail, mucoadhesion consists of: (1) establishing a contact between the mucoadhesive polymer and the mucus, which is influenced by attractive forces (i.e., electrostatic attraction and van der Waals interactions) and repulsive forces (i.e., osmotic pressure and electrostatic repulsion); this called the ‘contact phase’; (2) physicochemical interactions between polymeric and mucosal chains, which reinforce the adhesive bond; this is referred to as the ‘consolidation stage’.

EVs derived from the intestinal microbiome can represent ideal mucophilic drug carriers, as they are intrinsically able to pass through the aseptic mucous layer of the colon by different routes, enter the border intestinal epithelial cells, interact with mucosal immune cells and the intestinal vascular system, and promote its wide and systemic spread. Zheng et al. demonstrated in a mouse model of colitis that EVs derived from the bacterium *Akkermansia muciniphila*, a mucophilic member of the gut microbiota, prevent disease symptoms by reducing mucosal damage and increase the expression of MUC2 [151]. The reparation of mucosal damage on colon tissue was also demonstrated using EVs derived from *B. acidifaciens* in a colitis mouse model, with a complete restoration of the mucus and gut microbiota balance [152].

Also, chitosan (CS) shows relevant mucophilic properties. CS is a linear copolymer of β-(1–4) linked 2-acetamido-2-deoxy-β-d-glucopyranose and 2-amino-2-deoxy-β-d-glycopyranose, with a cationic nature [153,154,155]. Its biodegradability and biocompatibility characteristics, coupled with ecological safety and low toxicity, provide this molecule with the adequate requirements for use in drug delivery [156]. CS presents a positive charge that improves its permeability and mucoadhesive properties by establishing complex ionic, hydrophobic, and hydrogen bonding interactions with negatively charged mucosal surfaces [149]. In particular, CS’s ability to tightly adhere to mucosal surfaces, such as gastrointestinal tract walls, is the result of the protonation of its amino groups under acidic conditions, and this allows the transient opening of tight junctions between epithelial cells, making it an ideal compound for gut drug delivery. This feature enhances drug absorption through the intestinal epithelial cells [157]. CS’s excellent degradability by colonic flora and enzymes makes it an ideal carrier for intestinal drug delivery; it protects the drugs during their passage along the inflamed gastrointestinal tract in IBD therapy [158,159]. Nanocarriers based on CS were also developed as drug delivery systems for colon cancer [160,161,162,163,164,165]. It was also prepared by coupling with alginate [166] or folate [167,168,169] to increase specificity or cell uptake. Due to its structure, which allows its entanglement with other molecules and/or its adsorbtion on (negatively charged) NP surfaces, CS has been used not only as a basic compound for the development of NPs, but also for the surface modification of NPs [22]. In Zhang S. et al.’s study [170], the use of CS/HA/gelatin hydrogel microspheres allowed the drug to cross the upper digestive tract without loss of material and to reach the location-specific release in the colon, driven by the presence of polysaccharides that were specifically degradable by colonic bacteria [171,172]. CS and Polyethylenimine (PEI), a polycationic polymer able to form a positive layer around negatively charged liposomes and to establish electrostatic interaction with the negatively charged mucosal layer, was also considered as a liposome coating for the improvement of drug delivery in colon cancer [173].

Electrical affinity with intestinal mucosa is also at the basis of the work by Zhang C. et al. [174], who developed an oral polyphenol nanoparticle by assembling dexamethasone sodium phosphate (DSP)-loaded poly-β-cyclodextrin with tannic acid (PDT). PDT is negatively charged; this feature allows a specific targeting mechanism, as it can electrostatically attract the positively charged inflamed colonic mucosa. It allows precise delivery of the drug at the inflammation site, thus mitigating systemic exposure and minimizing potential side effects. A nanoparticle-assembled fluid coacervate was developed by Zhao et al. [175]; it showed good mucoadhesion on the luminal surface of porcine intestines, which was ascribable to the physical interactions between the catechol/PEG structure of coacervate and glycosylated mucins.

An original approach developed for IBD-targeted therapy involves the exploitation of hemoglobin nanoparticles (HbNPs) [176]. HbNPs have been explored as promising 5-ASA drug delivery carriers due to their naturally controlled degradation mechanisms and their relatively biocompatible and non-antigenic properties. The results showed that in the in vivo imaging of mice, the drug localized in the descending colon, following oral administration. The effectiveness of such NPs is based on the capability of hemoglobin to adhere to the mucus of intestinal epithelial cells. Wang et al. showed that the proteolytic enzymatic activity towards hemoglobin stops when it binds to NPs because its active sites are shielded from degradation [54,177]. The ability of HbNPs to increase the concentration of 5-ASA in the colon resembles that of other amino acids, such as aspartic acid, taurine and glycine, which are defined as ‘colon specific carriers’ [178,179]. The strong mucoadhesive properties of HbNPs allow the distal colon to be reached and the drug to be released slowly, resulting in an effective anti-inflammatory action in the Caco-2/HT29 cells [180]. Furthermore, the nanosized formulation (HbNPs) fosters the accumulation of particles at the inflamed site and mediates a transcytosis process in endothelial cells [181].

To summarize, mucophilic properties, which allow drug carriers to establish a reinforced contact with the intestinal mucus, are crucial to the support of targeted drug release to the gut. In particular, CS presents such a characteristic and is widely used as a nanocarrier, either alone or coupled with other compounds. Interestingly, even hemoglobin and EVs have mucoadhesive features and can enable effective drug delivery strategies in the gut (Figure 6(2)).

### 3.4. Release by Degradation Due to Colonic Flora or Intestinal Enzymes

Intestinal microflora enrichment presents wide variations among individuals and depends on multiple factors (e.g., diet, age, health condition, weight, exercise activity, etc.) [182]. It consists of hundreds of different species of bacteria which contribute to gut immune function, mucosal barrier activity, metabolism of drugs, and production of short-chain fatty acids and vitamins.

Numerous natural polysaccharides exhibit stability in the gastrointestinal tract, whereas they undergo biodegradation in the colonic microenvironment due to the activity of colonic flora and enzymes. This property represents a useful feature for applications in targeted delivery to the gut [183]. For example, pectin (PEC), a natural soluble complex polysaccharide [184] recognized as a dietary fiber [185,186], exhibits stability in the gastric tract but undergoes biodegradation in the intestine due to the presence of colonic flora and enzymes which promote fiber fermentation and separation in monosaccharides. Jing and colleagues prepared a colon-targeting system involving the preparation of PEC/Ca^2+^ microspheres, crosslinked by oligochitosan. The drug release profile was pH-dependent and responsive to the colonic microenvironment, showing a preferential distribution in the colon [187]. A form of PEC (the modified citrus pectin) that is able to bind galectin-3 and is highly expressed on the membranes of colon cancer cells was exploited to functionalize calcium phosphate NPs [188] and CS NPs [189].

Biopolymers such as xanthan gum, locust bean gum, and portulaca-derived polysaccharides have been used for the fabrication of smart microspheres [190,191,192]. Although these natural polysaccharides show resistance in the passage through the stomach and the small intestine, they are typically degraded by the anaerobic microflora of the colon. Katira gum, a polysaccharide of natural origin containing l-rhamnose and d-galactose sugar units and a small percentage of d-galacturonic acid, was investigated to improve carrier resistance in the colon, thus prolonging drug release time with better performances with respect to other gums and gels [193,194,195].

Elmorshedy et al. [196] developed calcium-crosslinked microparticles using inulin, carboxymethyl cellulose, and zein (a prolamin protein); their aim was to protect their payloads (lactoferrin) from degradation in the upper gastrointestinal tract. Release was allowed by inulin, which can be broken down by enzymes produced by resident colonic bacteria. Inulin was also successfully used for colon cancer drug delivery when coupled with HA nanoparticles [197].

Similarly, the lipase-induced degradation process occurring in the gut was exploited to effectively administer gene therapy in celiac disease [198,199]. DNA was encapsulated in nanoparticles in microsphere oral systems (NiMOS) represented by specifically engineered biodegradable and biocompatible polymer-based microparticles of poly(epsilon)-caprolactone matrix, which undergo degradation in the gut [200]. Such NPs can be endocytosed by the intestinal cells to undergo DNA transfection and expression.

Among different biocompatible motifs, a dextran coating was used for nano-assemblies [201] and SPIONs to protect the drug in the stomach and small intestine while promoting its release in the colon, as mediated by the presence of dextranase, an enzyme secreted by intestinal bacteria [202]. For the same purpose, Wang et al. coated mesoporous silica NPs with dextran [203]. An interesting case of intestinal therapy mediated by microflora involves the exploitation of bacterial EVs (BEVs) in IBD. The complexity of dysbiotic gut microbiota in IBD is responsible for the driving of aberrant immune activation and inflammation in the gut due to BEVs being released into the intestinal lumen. In particular, UC is characterized by a reduction in the quality of bacteria, and CD is characterized by an altered flora composition, especially with regard to Bacteroides and Firmicutes species [204]. The administration of Bacteroides thetaiotaomicron, one of the major Gram-negative anaerobe constituents of the human cecal and colonic microbiota, was shown to reduce inflammation in the colon lumen, which was, at least in part, mediated by its production of BEVs [205].

To summarize, the gut specific flora creates a typical environment that can promote the degradation of carriers in this site (Figure 6(3)). This is true, for example, for natural compounds such as pectin, inulin, gums, and dextran, all of which are decomposed by bacteria or enzymes specifically present in the intestine.

### 3.5. External Stimuli-Driven Release

Physical stimuli include a set of approaches such as ultrasound, heat [206,207], light, magnetic fields, or electrical and mechanical triggers. Some of them have been exploited for a long time in therapy delivery, such as in brachytherapy (or internal radiation), where radioactive material is placed inside the body to counteract cancer growth. More recently, such stimuli have been coupled to nanocarriers, thus enabling new types of drug delivery, which improve the specificity of release in the desired body region. This is the case, for example, with colon cancer treatment through liposomes, where their dissolution can be activated by external stimuli, such as a magnetic field or light, when they reach the correct location for drug delivery [208,209,210,211]. As already reported, SPIONs magnetize in response to an external magnetic field. This property makes the SPION another good candidate for therapies targeted through external stimuli. In the context of gut diseases, SPIONs are often exploited in colon cancer therapy [212]. SPIONs are typically composed of iron oxide [213,214,215,216,217,218] or cobalt-nickel-niobium-iron oxide [219,220] cores, and, as with other nanocarriers, they can be functionalized with (or encapsulated in) biopolymers which improve their biocompatibility and—in the context of gut-specific therapies—their interaction with intestinal mucosa. Specifically, colon tumor cells can be tackled by exploiting SPION-enabled molecular targeting (e.g., HA functionalization to bind CD44-overexpressing cells) or pH-promoted release [126,221]. Other than their straightforward application, SPIONs can, interestingly, be employed in hyperthermia upon stimulation with an alternating magnetic field. At specific frequencies they absorb electromagnetic energy, increasing their temperature, and the generated heat can be transmitted to targeted cancer cells [222]. NP functionalization with cell-penetrating peptides, such as the TAT cell-penetrating peptide [209] combined with magnetic navigation, improved the cell internalization of magnetic liposomes for hyperthermia treatments. EVs can also take advantage of external stimuli to improve their effectiveness. Modified EVs derived from goat milk turned out to be a great platform for photothermal enhanced therapy (PTT) for colon cancer [223,224]. Similarly, EVs derived from macrophages or cancer cells were loaded with photosensitizer zinc phthalocyanine (ZnPc), which increases the effectiveness of phototherapy in these treatment strategies with the promotion of immunological memory [225].

To summarize, external stimuli can force the delivery of a drug to the desired site. In this scenario (Figure 6(4)), the release is not modulated by chemical activity but by physical forces (such as ultrasound, heat, light, magnetic or electrical fields, or mechanical triggers), which move or activate carriers, like SPIONs.

### 3.6. Molecular Targeting

Nanocarrier functionalization is the most common strategy used to specifically target cells through their receptors. This approach is typically exploited in the cases of inflammation or cancer, where cells are known to overexpress some membrane proteins. A non-exhaustive list is reported in Table 2.

The most recognized target in this context is CD44. It is a ubiquitous transmembrane glycoprotein which acts as a receptor and regulates several interactions between cells and the extracellular matrix. Its isoforms are predominantly overexpressed in inflammation and cancer [22,226]. HA presents high affinity for CD44, in addition to pH-responsiveness. Therefore, a HA coating in NPs markedly enhances their cellular uptake by means of HA–CD44 interaction, which facilitates nanoparticle internalization through receptor-mediated endocytosis. A human antibody fragment, AbD15179, which is well characterized for its specificity for human CD44v6, an alternatively spliced CD44 isoform known to be overexpressed in 50% of colorectal cancers, was exploited for drug delivery in colon tumors [227]. Similarly, CD47 [228] and the mannose receptor (CD206) [229,230], overexpressed on the surface of colorectal carcinoma cells, were used as targets for the efficient targeting of colon cancer therapy.

The epidermal growth factor receptor (EGFR or HER1) is a membrane protein that is highly overexpressed in cancer. Its molecular targeting often enhances drug antitumor activity [231]. The epidermal growth factor (EGF), due to its specific and strong binding to the receptor, was successfully used as a functionalization strategy in colon cancer drug delivery [232,233], by exploiting, for example, PLGA nanoparticles [234] or lipid-based systems [235,236]. Liszbinski et al. proved that gold carriers coated with anti-EFGR stopped cancer cells [237]. Like its natural ligand EGF, the GE11 peptide also showed a good binding affinity to EGFR [238]. Even the human epidermal growth factor receptor 2 (HER2) represents a valuable target for colon cancer drug delivery as it is overexpressed in some colon cancer patients [239].

Folate receptor, a glycosyl-phosphatidyl-inositol anchored on the cell surface, is overexpressed in most cancers, enabling their targeting by functionalization of the nanocarrier with folic acid (FA) [240,241,242,243,244,245]. Functionalization with FA improved the specific delivery to cancer cells and also had a combined chemo/magnetothermal therapeutic effect [90,202,246,247,248,249,250,251]. FA was employed as the main targeting ligand in the development of a drug delivery system for colchicine, which is highly cytotoxic. The delivery system consisted of the use of mesoporous silica nanoparticles coated with an FA-CS-glycine complex [252].

Some groups tried to bind nucleolin, a protein highly expressed in the surface of colorectal cancer cells. Babaei et al. designed PEGylated mesoporous silica nanoparticles as a vehicle for the co-delivery of camptothecin and survivin shRNA-expressing plasmid. The system was functionalized with AS1411, a 26-base Guanine-rich DNA aptamer which presents high affinity and specificity in the external domain of nucleolin [253,254,255,256]. This formulation suppressed the tumor growth rate in colon cancer cells and tumor-bearing mice [257]. Analogously, AS1411 was used to functionalize NPs of albumin, a water-soluble endogenous protein which shows biocompatibility, non-toxicity, low immunogenicity, and biodegradability properties, thus presenting great drug-binding capacity [258].

A set of other functionalizing molecules were exploited to target colon cancer cells based on overexpressed membrane proteins. Carrier surface decoration with Urotensin (UT) increases the inhibition of cancer cells overexpressing UT receptors [259]. VATANST peptide (STP) emerged as specific for colon cancer targeting since it binds to vimentin, which is highly expressed on the surface of colon cancer cells [260]. Glucocorticoids were used as functionalization molecules to enhance colon cancer targeting since they show efficient and selective cytotoxicity in colon cancer [261]. Other peptide receptors (e.g., leptin-derived peptide (Lp31) [262] are known to be expressed in different type of cancers. FA12 peptide and AR13 peptide are used for targeting cancer cells overexpressing Muc1 [263,264]. Ligands such as Tumor Necrosis Factor-Related Apoptosis-Inducing Ligand (TRAIL) [265], Frizzled 10 (FZD10) Protein [266], EpCAM Aptamer [267], Programmed Cell Death Protein 1 (PD-1) [268], RGD [269], CC-9 and RH-20 [270], and specific DNA sequences [271] have demonstrated enhanced uptake by cancer cells and cytotoxicity.

Gut drug delivery systems can be achieved by conjugating carriers with wheat germ agglutinin (WGA), which binds specifically to the cell membrane of colon cells. WGA is a moiety for active targeting because of the binding to gastrointestinal mucosa through the recognition of N-acetylglucosamine and sialic acid residues on the cell membrane, thus leading to cellular internalization through receptor-mediated endocytosis. Moreover, it is resistant to acidic pH and enzymatic degradation [272], therefore, it is being extensively used as a functionalization molecule in gut-specific targeting. WGA was used to develop nanocarriers to target gut cells based on thiolated alginate [273] and sodium alginate [79]. A similar strategy was pursued by Wang et al. [272], who conjugated colon cancer drug delivery nanoparticles made of gelatin and CS with WGA.

The gut-specific bacteria-guided targeting approach has great potential, especially in cancer treatment. The intravenous administration of bacteria-targeted silica nanoparticles functionalized with bacterial lipoteichoic acid (LTA) antibody was exploited to deliver colon antitumor drugs [274,275].

A strategy commonly exploited in drug delivery for IBD treatment involves the targeting of macrophages, which are closely involved in the inflammatory process related to this and several other gut pathologies and are therefore being considered as attractive target cells [22], to maximize the delivery in the desired locus and to minimize the systemic side effects of anti-inflammatory drugs. Although targeting macrophages represents a widespread approach for reaching affected colon tissues, it is a method which is too general-purpose with respect to the aim of the present review; therefore, it was excluded from this dissertation. Recently, a complete cancer eradication was achieved with a vaccine based on a neoantigen-painted serum exosome in combination with programmed cell death protein 1 (PD-1) antibodies in mice with colon cancer, opening a new scenario involving the use of exosomes derived from patients and enhanced with directional antibodies for highly efficient personalized therapy [276].

To summarize, molecular targeting should represent, at least in principle, the most specific method for site-directed drug release. This is valid for colon cancer, where a number of membrane proteins are known to be overexpressed (e.g., CD44 and its isoforms, folate receptors, HER1, and nucleolin). For other gut-affecting pathologies, no effective molecular targeting exists, except for WGA, which is known to bind specifically to the cell membrane of colon cells.

### 3.7. Uptake-Enhancing Strategies

Specific molecules appeared to enhance drug uptake in the design of nanocarriers, by improving either drug stability (as in the case of PLGA) or bioavailability (such as for polymeric micelles), or to increase drug absorption in tissues (such as in the case of folic acid), or to improve the residence time in the tissues, leading to augmented therapeutic activity.

Poly(lactic-co-glycolic) acid (PLGA, CAS: 26780-50-7) is a biocompatible polymer approved by the FDA. Despite its anionic nature, which gives it poor mucoadhesiveness (both the gastrointestinal mucus and PLGA present negative charges) [149], several nanocarrier solutions for drug delivery in the gut involve its use. In fact, PLGA was proven to have low immunogenicity, hydrophobicity, biocompatibility, and biodegradability. It was demonstrated to have high efficiency and versatility in the encapsulation of both hydrophilic and hydrophobic factors [277]. It enhances drug stability (protecting it from degradation) and oral bioavailability, ensures the drug’s prolonged release, and increases its concentration at the site of inflammation, reducing the side effects of toxic molecules. It also enables efficient delivery of water-soluble compounds, which, without encapsulation, would be repelled by the limited permeability of the mucosal membranes, leading to rapid clearance of the drugs [149]. In addition, PLGA is degraded in the body into nontoxic lactic acid and glycolic acid, which are the normal components of Kreb’s cycle that are subsequently eliminated as carbon dioxide and water, without affecting the normal cellular functions [278]. However, the PLGA nanocarriers; targeting ability does not appear to be totally reliable, potentially causing systemic drug delivery [279]. To overcome this problem, methods to improve targeting ability were tested, such as coating PLGA NPs to counteract their charges, thus promoting electrostatic interactions with negatively charged mucous and cellular membranes, or combining PLGA with other polymers. For example, by using functional polymers like poly(ethylene glycol) methyl ether-block-poly(lactide-co-glycolide) (PLGA-PEG), mucus-penetrating properties can be gained [278]. PLGA was exploited to enhance the performance of IBD treatments [280,281,282,283], including the case of mucositis, an inflammatory condition of the intestinal mucosa [284], and in colon cancer therapy [285,286,287,288,289,290,291]. Naserifar et al. designed a drug delivery system consisting of folic acid-conjugated PLGA NPs to deliver resveratrol to intestinal cells [292]. Folic acid is known to initiate caveolae-mediated endocytosis, thus helping to improve tissue absorption and bioavailability of the drug [293]. The therapeutic efficacy was evaluated in a colitis rat model which considered the remission of colon inflammation. In in vitro permeability experiments, the NP system passed the Caco-2 cell monolayer in a significantly enhanced manner with respect to free resveratrol.

More recent drug delivery systems consist of nanoplexes, niosomes, and polymeric micelles. The former present several advantages over other NP systems, especially in the preparation, which involves mixing only drug(s) and polymer solutions, and it requires minimum energy and does not necessitate the use of sophisticated equipment [294]. Niosomes consist of layered structures of vesicles made from non-ionic surfactants and can be modified to be magnetically driven, as exploited even in the context of colon cancer [295]. Polymeric micelles are aggregation colloids formed in solution by the self-assembling of amphiphilic polymers. Micelles represent a novel drug vehicle; with respect to other nanocarriers, polymeric micelles generally have a smaller size, present easier preparation and sterilization processes [296], and allow high bioavailability of poorly water-soluble drugs [297]. Thanks to their good solubilization properties, micelles appeared to help in IBD [121] and colon cancer treatments [298,299], with notable advantages, such as reduced risk of systemic toxicity, improved selectivity for specific tissues due to stimuli-sensitive polymeric materials, incremented storage stability, and resilience against dilution. Xu et al. designed a micelle-based oral nanoparticle formulation formed by curcumin-conjugated hydroxyethyl starch. They produced dexamethasone (DEX)-loaded NPs with desirable size, negative surface charge, good stability in the harsh gastric environment, and excellent ROS scavenging activity [299]. For colon cancer, polymeric micelles are also employed. For instance, Banskota et al. developed ‘zwitterionic polypeptides’ that served as ‘stealth’ vehicles for drug delivery across various tumor types, with a particular emphasis on cancer cells [300].

Lipid-based nanocarrier formulations have been recognized as a useful strategy in drug delivery due to their effectiveness in prolonging the colonic passage of drugs. Lipid systems were used to enable drug targeting in IBD [301,302,303] and in colon cancer [304,305,306], such as in the form of FA-conjugated liposomes [240]. The liposome encapsulation of chemically unstable compounds can offer a physical barrier that prevents the oxidative degradation of these compounds [307]. Interestingly, liposome-based delivery methods have been developed to also release mRNA [308] and siRNA molecules [309] in the gut, in addition to the typical anti-inflammatory drugs [310]. A lipid–polymer hybrid nanoparticle system was designed to deliver the antioxidant superoxide dismutase (SOD) [311], an enzyme which can undergo hydrolysis and enzymatic degradation due to the harsh conditions in the gastrointestinal tract. The lipid carrier preserved the enzyme integrity, while the poly(ethylene glycol) (PEG) and folate functionalization surface coating of the lipid–polymer hybrid nanoparticles allowed the delivery in the adequate tissue; this was also the case in in vivo experiments, where it showed excellent mucus-penetrating ability and inflammation-targeting properties. In some cases, liposome functionalization can exploit the different cytotoxicity levels of compounds according to their ionization state, thus enhancing targeting ability. In the case of malachite, for example, acid conditions promote the conversion from the neutral low-toxic form to the cationic cytotoxic state, thus improving tumor cell killing selectivity [312]. Recently, NLC systems were developed in the context of drug delivery to the gut and were able to improve drug loading and stability [313]. The oral administration of curcumin encapsulated in solid binary lipid NPs increased stability and uptake in IBD; the efficiency of these types of NLCs was due to the small dimension range (210 ± 41.22 nm) and the high entrapment efficiency (83.12 ± 6.57%) [314]. NLC systems showed high toxicity against colon cancer cells, and optimal cell internalization [315,316] and also demonstrated good properties in patient administration [317].

As with liposomes, nanocubosomes are emerging as powerful nanocarriers due to their inherent potential to encapsulate both hydrophilic and lipophilic drugs into isotropic cubic liquid crystalline nano-systems with a large interior surface area. They have been tested, for example, in colon cancer drug delivery [318].

Exosomes and, more generally, EVs also represent, especially in the context of cancer [319,320], a useful approach for targeted drug delivery since they are intrinsically targeted vehicles. In fact, they naturally contain [321], or can be forced to contain (engineered exosomes) [322,323], therapeutic molecules. Modified tumoral EVs were used to increase the uptake of Napabucasin (NAP) by loading it in the EVs and injecting in affected mice, showing an increased anti-tumoral and anti-inflammatory action of NAP with respect to free drug administration [324]. A novel strategy for drug delivery consists of herb-derived exosomes. A large mesoporous silicon nanoparticle was constructed using a ginger-derived exosome and an inorganic framework for the oral delivery of infliximab as IBD therapy. These nanoparticles could target the colon and are taken up by epithelial cells and macrophages. However, the molecular mechanism of nanovesicle colon targeting is still unclear [325]. Zhang L. et al. [326] designed a colon-specific delivery system composed of micro- and nano-encapsulated hybrids (MNEHDS) for oral delivery of Berberine and tested its therapeutic efficacy in a murine colitis model. Membrane coating nanotechnology has recently been implemented to improve nanoparticle biocompatibility, biodegradability, long-term circulation time, and immune escape ability. Using this cell-mimetic strategy instead of synthetic strategies, the intrinsic ability of cells to interact with physiological environments can be exploited, thus improving the interface between the nano-delivery systems and the target site. Biomimetic nanocarriers were fabricated by coating NPs with cell-derived membranes; thanks to their easy extraction and large availability, erythrocytes were used to enhance drug delivery in colon cancer [327,328]. He et al. developed gold nanoparticles with a peptide macrocycle inhibitor (PMI) that inhibits different proteins involved in colon cancer cell activity, such as MDM2 e MDMX [329,330].

Recently, SEDDS/SNEDDS/SMEDDS were used to improve the stability, solubility, intestinal permeability, and bioavailability of lipophilic compounds in the gut, which present poor solubility in water and result in low bioavailability of the drug. The exploitation of such systems seems to widely improve the targeted and effective release of drugs to the gut [331,332,333,334,335,336,337].

## 4. Discussion and Conclusions

Targeted drug delivery enabled by nanocarriers proved to be essential for several reasons, such as the promotion of the specificity of the treatments in a wide range of pathologies, the reduction in off-target delivery and related collateral effects, and the modulation of drug release time. Moreover, the exploitation of drug carriers helps in the maintenance of drug stability [338].

The aim of this review is to provide the reader who is approaching the topic of drug release in the gut with an overview of the current targeted delivery strategies. To this end, the literature analysis relied on the selection of the most extensively used nanocarriers and of a set of pathologies with a high impact on the intestine. The systematic approach to the literature allowed the unbiased identification of several methods that are used to enable the intestinal delivery of therapies. Several NPs that were able to target the gut emerged, spanning from organic (e.g., liposomes) to inorganic (e.g., SPIONs) types and from those of natural (e.g., EVs) to those of synthetic (microspheres) origin; these NPs either acted as drugs themselves or were exploited as carriers for the delivery of other substances. To better focus on the therapy release methods, the retrieved papers were classified based on the ‘driving force’ which guides drug nanocarriers to the gut. This led to the identification of five major intestine delivery strategies (Figure 6): (1) pH responsiveness; (2) mucophilic properties; (3) release mediated by colonic flora or intestinal enzymes; (4) external stimuli-driven release; and (5) molecular targeting. In fact, most methods are tightly related to the biological/physical/chemical peculiarities of the gut, namely the ‘gastrointestinal pH’, the ‘gastrointestinal mucus’, and the ‘gastrointestinal enzymes and microbiome’ [3,338]. Such features allow the development of ‘passive’ delivery strategies [3], which do not rely on specific ligand target bindings.

pH responsiveness appears to be a recurrent method to deliver drugs to the gut, given the peculiar pH variations that are intrinsic to the gastrointestinal tract in pathophysiological conditions. pH responsiveness is expressed by several polymers, such as alginate/sodium alginate, HA, and eudragits and also by silica particles [76,77,78,109,139,140,339]. In particular, HA seems to be a highly versatile molecule for gut targeting, being not only pH sensitive but also the natural ligand for CD44 receptor, which is overexpressed in several gut pathological conditions, such as colorectal cancer. The advantage of using pH-responsive carriers is that they allow the protection of drugs during their passage through the acid environment of the stomach, whereas they are released in the intestine.

Another set of ‘passive’ gut-targeting strategies relies on the mucophilic property shown by molecules such as CS and hemoglobin, which can strongly interact with intestinal mucus. Such molecules are widely used as basic structures for nanocarriers or to functionalize them, reinforcing the strategy for targeted drug delivery to the gut [176]. Mucoadhesive carriers are used to improve intestinal adhesion, prolonging contact between the released drug and the intestinal mucosa to finally improve drug absorption. For this reason, their exploitation can be advantageous in the presence of the pathologies that cause increased colonic motility, diarrhea, and, consequently, reduced retention, such as IBD [338].

The enrichment of colonic flora and specific enzymes plays an important role among the strategies for targeted drug delivery to the gut due to their ability to selectively degrade materials. For this reason, polysaccharides such as pectin, inulin, and some natural gums, which can resist the passage through the gastric tract but are degraded by the intestinal microbiome, were largely used as nanocarriers [127,184,187,188,189,196,197]. Drug release based on resident bacteria seems to represent a common strategy, although the inflammatory condition related to several gut pathologies, especially when associated with severe diarrhea, can affect the composition of gut microflora [340]. An interesting approach for intestinal therapy mediated by microflora involves the exploitation of bacterial EVs. EVs are able to confer stability to drugs and can direct their cargoes to specific cell types [341]. In particular, bacterial EVs are produced and released by resident bacteria to communicate with host cells and other bacteria and show easy uptake by host cells [342]. For this reason, EVs can represent a smart and futuristic type of nanocarrier, which can be used either as naturally produced by the organism (in the case that they intrinsically contain the desired molecule) or as engineered, to load exogenous therapies [54,59,60,61]. Nevertheless, the lack of standardized protocols for the purification and isolation of such BEVs currently represents a limit to their usage [342].

Molecular targeting, which represents the only ‘active’ targeting method and typically allows the highest specificity in selective delivery [3], shows moderate applicability in the gut context. Actually, most of the ligand-based intestinal carriers were developed for the condition of colorectal cancer. In such a disease, several membrane proteins are known to be overexpressed and can be used as targets to enable specific drug delivery. This is the case with CD44, EGFR, or the folate receptor, among others [22,226,231,240,241,242,243,244,245] (Table 2). The only strategy that seems to allow specific binding to the gut epithelium, independently of its physiological or pathological condition, involves WGA. WGA is extracted from wheat germ. Being a N-Acetylglucosamine-binding protein, it specifically binds to the sugars expressed, among many others, by the human gastrointestinal epithelial [343]; for decades, it has been known to be able to traverse the human small intestine intact [344]. For this reason, it has been exploited to functionalize nanocarriers to improve their gut specificity [79,272,273,345], although, overall, the gut does not represent its unique target [345].

Finally, instrumental modes can allow drugs to reach the gut, taking advantage of physical stimuli. In these cases, cues such as the magnetic or electric field, light, heat, or other forces can either move drugs to the gut or enable nanocarrier dissolution and drug release when they reach the desired site [229,230,253,254].

As already reported, each gut-targeting strategy shows peculiar advantages and/or adequateness with regard to the specific gut condition (such as cancer or inflammation). It is worth noting that many proposed carrier solutions adopt multi-purpose strategies, coupling together materials that can enable different gut-targeting methods. Some interesting examples include the following: (1) alginate and CS are often used together, to guarantee that nanocarriers have the features of pH sensitiveness and mucoadhesion [89]; (2) alginate can be coupled to folic acid to provide the possibility to not only enable drug release in the colon but also to specifically target cells overexpressing folate receptors [90]; (3) eudragit assembled with sodium alginate and inulin has the capacity to express both pH sensitivity (through eudragit and sodium alginate) and drug release by means of intestinal microbiome [127]; (4) as already reported [105,106], HA alone can intrinsically trigger two different types of gut affinity (through pH sensitivity and molecular binding to CD44); thus, it is an optimal compound in targeted drug release, especially in cancer or IBD therapy.

Other considerations emerge from this extensive review. The first concerns the fact that the large majority of therapies targeting the gut are administrated through the oral route. This has the clear advantage of being non-invasive. Moreover, it exploits the natural passage of substances along the whole gastrointestinal tract; thus it is intrinsically directed towards the desired drug release site [338]. Additionally, oral administration can take advantage of the gut’s peculiar conditions, such as pH level and the presence of mucosa and colonic flora, which can support drug release specifically to the gut. On the other hand, drug passage along the whole gastrointestinal tract represents a challenge for the stability of the therapy, which encounters harsh environments. Moreover, oral administration may prove ineffective in the presence of severe dysmotility, such as in the case of chronic intestinal pseudo-obstruction (CIPO) [346,347], especially in acute scenarios. In such conditions, other administration modes (such as the intravenous one, coupled with different drivers for drug release) could result in being more effective.

The second important aspect involves the use of preclinical models. Most of the targeting mechanisms were tested in vitro. Co-culture and animal tests were additionally performed to ensure the uptake in the desired site/cells, often relying on models of murine origin. Considering the intrinsic anatomical and pathophysiological differences between human and mouse gut, more efforts are needed to improve the translational value of both exploratory research and preclinical studies, moving towards human-relevant engineered gut models [348].

A final consideration concerns the carriers’ toxicity. In fact, nanocarriers present critical features, such as particle size, which allows easy access to organs, and a charged surface, which increases the chances of non-specific interaction [349]. Most compounds used as drug carriers show high biocompatibility and are approved by the FDA. Nevertheless, for some nanocarriers, toxic effects have been highlighted. In this regard, metal-based NPs, for example, were associated with increased oxidative stress and seemed to infiltrate the cell nucleus [349]. Some studies highlighted the toxicity of silica nanoparticles, which is often dependent on dose [35]. The major concern in the use of liposomes involves their loss of stability in the presence of lipoproteins in the blood, which causes the rearrangement of the lipid surface. To overcome this condition, liposomes often undergo PEGylation. Regarding polymeric NPs, natural polymers should be used as potential alternatives to synthetic polymers to increase biocompatibility. Several studies report the toxicity of SEDDS, and their disruptive action on intestinal microbiota, which is correlated with the increased expression of proinflammatory cytokines, the reduction in plasma citrulline levels, and the reduction in the mucosal barrier integrity [350]. In this regard, Naimi et al. (2021) underlined the negative impact of some synthetic emulsifiers on the composition and function of intestinal microbiota, with pro-inflammatory molecule production and the consequent increase in intestinal inflammatory syndrome [351]. Analogously, Subramaniam and colleagues highlighted the fact that some synthetic excipients cause dysbiosis, as opposed to natural compounds (such as starches, gums, and alginate) that increase microbiota diversity and abundance [352].

In conclusion, the strategies for targeting the gut in drug release are attracting attention, as shown by the huge number of publications in the field. This could be ascribed to the impact that some not yet completely solved intestinal pathologies have on the population (such as colorectal cancer, IBD, IBS, and celiac disease) and to the cruciality of this multifunctional organ for the quality of life. Although targeted delivery represents a major need for the improvement of the effectiveness of therapies for intestinal pathologies, the identification of organ-specific or, even better, tissue-specific release mechanisms is still challenging. The overall environment of the colon, which presents specific pH, extended mucus, and peculiar microflora, can support several strategies that use ‘passive’ targeting. On the other hand, according to the best current knowledge, it seems that most of the ‘actively targeted’ ligands are typical of specific pathologies rather than of the gut; active targeting can be performed only in the presence of cancer and inflammatory conditions, for which overexpressed cell membrane proteins have been clearly identified.

## Figures and Tables

**Table 1:** Number of queried papers, ‘pre-’ and ‘post-’ application of the exclusion criteria.

	*SPIONs*		*Liposomes*		*Silica*		*“Extracellular Vesicles” and “Therapy”*		*Nano-Delivery Systems*		*Nanoparticles and “Drug Delivery”*		*Microspheres*		*NLCs*		*SEDDS/SNEEDS/SMEEDS*	
***Exclusion process***	*pre*	*post*	*pre*	*post*	*pre*	*post*	*pre*	*post*	*Pre*	*post*	*pre*	*post*	*pre*	*post*	*pre*	*post*	*pre*	*post*
***IBD***	0	0	32	9	16	1	45	5	6	5	105	29	17	5	14	1	6	3
***Colon cancer***	4	3	85	35	60	36	45	7	5	1	227	140	16	8	41	7	4	3
***Celiac disease***	4	1	0	0	1	0	5	0	1	0	18 *	3	3	0	0	0	2	1
***IBS***	0	0	1	0	1	0	20	0	0	0	9 *	3	3	2	4	0	0	0

* In order to increase the number of outputted results for IBS and celiac disease, the ‘Drug delivery’ term was left out of these queries.

**Table 2:** Summary of the receptors overexpressed in colon cancer, useful in targeting affected cells.

Overexpressed Receptors	Specific Ligands
CD44	HA
CD44v6	AbD15179 (antibody fragment)
CD206 (mannose receptor)	Mannose
Nucleolin	AS1411
EGFR (or HER1)	EGF and GE11
Urotensin receptor	Urotensin
Folate receptor	FA
Vimentin	VATANST peptide
Muc1	FA12 peptide and AR13 peptide
